# The Transcription Factor CsAtf1 Negatively Regulates the Cytochrome P450 Gene *CsCyp51G1* to Increase Fludioxonil Sensitivity in *Colletotrichum siamense*

**DOI:** 10.3390/jof8101032

**Published:** 2022-09-29

**Authors:** Xiaoling Guan, Miao Song, Jingwen Lu, Hong Yang, Xiao Li, Wenbo Liu, Yu Zhang, Weiguo Miao, Zhigang Li, Chunhua Lin

**Affiliations:** 1 Key Laboratory of Green Prevention and Control of Tropical Plant Diseases and Pests, Ministry of Education, College of Plant Protection, Hainan University, Haikou 570228, China; 2 Rubber Research Institute, Chinese Academy of Tropical Agricultural Science, Haikou 571101, China

**Keywords:** *Colletotrichum siamense*, CsAtf1, *CsCyp51G1*, RNA-Seq, ChIP-Seq, fludioxonil sensitivity, cytochrome P450

## Abstract

Previous studies have shown that the high-osmolarity glycerol mitogen-activated protein kinase (HOG MAPK) signaling pathway and its downstream transcription factor CsAtf1 are involved in the regulation of fludioxonil sensitivity in *C. siamense*. However, the downstream target genes of CsAtf1 related to the fludioxonil stress response remain unclear. Here, we performed chromatin immunoprecipitation sequencing (ChIP-Seq) and high-throughput RNA-sequencing (RNA-Seq) to identify genome-wide potential CsAtf1 target genes. A total of 3809 significantly differentially expressed genes were predicted to be directly regulated by CsAtf1, including 24 cytochrome oxidase-related genes. Among them, a cytochrome P450-encoding gene, designated *CsCyp51G1*, was confirmed to be a target gene, and its transcriptional expression was negatively regulated by CsAtf1, as determined using an electrophoretic mobility shift assay (EMSA), a yeast one-hybrid (Y1H) assay, and quantitative real-time PCR (qRT-PCR). Moreover, the overexpression mutant *CsCYP51G1* of *C. siamense* exhibited increased fludioxonil tolerance, and the *CsCYP51G1* deletion mutant exhibited decreased fludioxonil resistance, which revealed that *CsCyp51G1* is involved in fludioxonil sensitivity regulation in *C. siamense*. However, the cellular ergosterol content of the mutants was not consistent with the phenotype of fludioxonil sensitivity, which indicated that *CsCyp51G1* regulates fludioxonil sensitivity by affecting factors other than the ergosterol level in *C. siamense*. In conclusion, our data indicate that the transcription factor CsAtf1 negatively regulates the cytochrome P450 gene *CsCyp51G1* to increase fludioxonil sensitivity in *C. siamense*.

## 1. Introduction

*Colletotrichum* spp. is considered one of the top ten plant pathogens and can cause devastation in a wide range of plants worldwide [1,2]. Moreover, *Colletotrichum* spp. is also a class of severe pathogens of tropical plants, such as rubber tree [3], mango [4], banana [5], and pepper [6]. *Colletotrichum siamense*, a member of the *Colletotrichum gloeosporioides* species complex, is a widely distributed phytopathogen that is recognized as the predominant pathogenic species causing rubber tree anthracnose in China [7,8].

Two-component regulatory systems are signal transduction systems that sense and respond to environmental changes. In eukaryotes, these systems usually consist of three activities: HK (histidine kinase), HP (histidine phosphotransfer), and RR (response regulator) activities [9]. As shown in previous studies, the signals can be transmitted via a multistep phosphorelay mechanism involving proteins such as Sln1 (HK), Ypd1 (HP), and Ssk1 (RR) [10], which in turn activates the high-osmolarity glycerol (HOG) mitogen-activated protein kinase (MAPK) cascade [11]. The HOG pathway, a branch of the MAPK signal transduction system, was first reported in fungi to coordinate the adaptation of yeast to elevated osmotic pressure [12], and subsequent experiments confirmed that this pathway is also involved in the oxidative stress response in *Saccharomyces cerevisiae* [13]. Although the regulatory mechanisms and molecular targets of the HOG MAPK pathway differ, this pathway has been reported to be involved in stress responses in almost all eukaryotes. Our previous study on *C. gloeosporioides* showed that the MAPKK *Pbs2* of HOG MAPK is involved in hyperosmolarity sensing and regulation of the resistance to fludioxonil [8].

Fludioxonil is considered the main representative phenylpyrrole fungicide [14]. It is generally agreed that fludioxonil can activate the HOG MAPK signaling pathway through group III HHKs over a long period of time [15]. Brandhorst et al. proposed that fludioxonil interferes with the activity of triosephosphate isomerase, which leads to the release of methylglyoxal, resulting in activation of the group III HHKs [16]. Because the HOG pathway is responsible for cellular adaptation to environmental stresses such as osmotic stress, it is generally accepted that the fungicidal activity of fludioxonil can be attributed to disorder in osmotic signal transduction [17,18]. Based on previous studies, Kilani and Fillinger speculated that the action mode of fludioxonil may involve simulation of osmotic stress by activating the HOG MAPK pathway [14]. In *Magnaporthe oryzae*, recent studies have shown that although both fludioxonil and osmotic stress can activate Hog1 of the HOG MAPK pathway and transport it to the nucleus, there are differences in their molecular mechanisms [19]. However, the specific mechanism of action of fludioxonil has yet to be identified.

The transcription factor *Atf1* belongs to the *ATF*/*CREB*-type bZip transcription factor family and functions downstream of the HOG MAPK pathway [20,21]. In *Aspergillus nidulans* and *Aspergillus fumigatus*, both *AtfA* (*Atf1*) protein activity and *atfA* (*Atf1*) gene transcription are controlled by the HOG MAPK pathway [22,23]. Phosphorylated SakA (*Hog1*) binds to *AtfA* in the nucleus, which is essential for the activation of genes regulated by *AtfA* in both *Aspergillus* species [24,25]. The functions of *Atf1* and homologous genes differ among various plant pathogenic fungi. Most studies have shown that *Atf1* homologues play a role in the regulation of the stress response and virulence [21,26,27,28]. Regarding its function in fludioxonil stress, it has been reported that HogA (Hog1 homology) and its downstream transcription factor *ATFA* are required for the transcriptional responses to fludioxonil in *A. nidulans,* but unlike the Δ*HogA* strain, Δ*ATFA* showed only slight resistance to fludioxonil [22]. Therefore, Hagiwara et al. suggested that the SskA-HogA-*ATFA* pathway is involved in the response to fludioxonil, but *ATFA* is not involved in the inhibitory effect of fludioxonil on growth [23]. However, our previous study in *Colletotrichum siamense* confirmed that the transcription factor CsAtf1 is involved in the fludioxonil stress response, and the Δ*CsAtf1* strain exhibited similar resistance as the reported deletion mutants of the upstream genes *Hog1* and *Pbs2* [28]. In view of the characteristics of transcription factors in the regulation of gene transcription, further research on the target genes downstream of CsAtf1 may be helpful for analyzing the mechanism underlying the regulation of fludioxonil sensitivity.

Cytochrome P450s (CYPs) constitute a group of monooxygenases that form a large and complex single gene superfamily involved in drug metabolism in organisms, including fungi [29,30]. Sterol 14α-demethylase (CYP51), a member of the P450 superfamily, is the most highly conserved and widely distributed in all biological kingdoms [31]. Most fungi contain one or multiple *CYP51* paralogues, such as *CYP51A1*, *CYP51A2*, *CYP51B*, *CYP51C*, and *CYP51D* [32,33]. It has been reported that *CYP51* genes in *S. cerevisiae* are overwhelmingly present as single genes [34], and in *A. fumigatus* and *Colletotrichum* spp., there are two homologous genes, namely, cyp51A and cyp51B [35,36]. However, *Aspergillus oryzae*, *Penicillium digitatum,* and *Fusarium* species have three paralogues of *CYP51* [31,37,38]. *CYP51* is reported to be the target of many fungicides and is associated with the resistance to fungicides, especially azoles [33,38,39]. Some of them were confirmed to be involved in pathogenicity, such as *Mocyp51A* in *Magnaporthe grisea* [40]. In *C. gloeosporioides*, *CgCYP51A* or *CgCYP51B* also play a role in the sensitivity to demethylation inhibitors (DMIs) [38]. A *CYP51* paralogue, *CYP51G1*, was reported to encode an obtusifoliol 14α-demethylase for phytosterol synthesis and affect pollen and seed development in rice [41]. However, the function of *CYP51G1* in fungi has not been reported.

In this study, on the basis of our previous studies [8,28], we asked whether the HOG MAPK CsAtf1 modulates the sensitivity to fludioxonil in *C. siamense*. Therefore, we further investigated the downstream target genes of CsAtf1 by chromatin immunoprecipitation sequencing (ChIP-Seq) and RNA sequencing (RNA-Seq), and the results revealed some CYPs to be target genes of CsAtf1. Among these CYP target genes, the sterol 14α-demethylase gene *CsCyp51G1* was identified as a target gene of CsAtf1 and was negatively regulated by CsAtf1 using an electrophoretic motility shift assay (EMSA), a yeast one-hybrid (Y1H) assay, and quantitative real-time PCR (qRT-PCR). We further investigated the phenotypes of *CsCyp51G1* overexpression and deletion mutant strains, and the results showed that *CsCyp51G1* was involved in fludioxonil sensitivity regulation. We propose the existence of a module via which the HOG MAPK pathway and the transcription factor CsAtf1 negatively regulate *CsCyp51G1* gene expression to increase fludioxonil sensitivity in *C. siamense*.

## 2. Materials and Methods

### 2.1. Strains and Culture Conditions

The *C. siamense* HN08 strain was used as a wild-type (WT) strain in this work. The mutant strains Δ*CsCyp51G1,* Δ*CsAtf1,* and Δ*CsPbs2*; overexpression strain *CsCyp51G1-OE*; *CsAtf1-GFP* fusion-expressing strain; and other related transformants were derived from HN08 in this study or constructed by our research group previously [8,28]. For collection of conidia, hyphae were placed on potato dextrose agar (PDA; 200 g/L potato, 20 g/L dextrose, and 20 g/L agar) and cultured under continuous fluorescent light for 3–5 d at room temperature. For extraction of DNA, RNA, and total protein, mycelia were cultured in liquid complete medium (CM; 0.6% yeast extract, 0.1% casein acid hydrolysate, and 1% sucrose) for 3–5 d in the dark at 28 °C.

### 2.2. Chromatin Immunoprecipitation Sequencing (ChIP-Seq) and RNA Sequencing (RNA-Seq) Analyses

Hyphae of the CsAtf1-GFP fusion-expressing strain were collected from a 5 d liquid CM culture and treated with 1% formaldehyde so that DNAs and proteins binding to each other were crosslinked and fixed. Samples were sent to IGENEBOOK Biotechnology Co., Ltd. (Wuhan, China) for coimmunoprecipitation, library building, and sequencing. Due to the lack of reference genomes and annotations for *C. siamense*, *Colletotrichum fructicola* was used as the reference [42]. Identification of peaks was performed using MACS2 [43] and ChIP-Seq results were visualized by Integrative Genomics Viewer [44]. HOMER (http://homer.ucsd.edu/homer/download.html, accessed on 5 June 2022) was used to predict sequence motifs of peak regions.

The HN08 strain and deletion mutant Δ*CsAtf1* were cultured for 5 d in liquid CM for transcriptome sequencing. The *C. fructicola* genome was used as the reference genome. The sequencing data was filtered with SOAPnuke [45] by (1) removing reads containing sequencing adapter; (2) removing reads whose low-quality base ratio (base quality less than or equal to 15) is more than 20%; (3) removing reads whose unknown base (‘N’ base) ratio is more than 5%, afterwards clean reads were obtained and stored in FASTQ format. The clean reads were mapped to the reference genome using HISAT2 [46]. Expression level of genes were calculated by RSEM (v1.3.1) [47]. Differential expression analysis was performed using the DESeq2 (v1.4.5) [48] with fold change > 2 and Q value ≤ 0.05. The gene expression levels were calculated using fragments per kilobase of transcript per million mapped reads (FPKM) values. Gene Ontology (GO) enrichment information for the differentially expressed genes was obtained by EasyGO (http://bioinformatics.cau.edu.cn/easygo/, accessed on 6 June 2022). Kyoto Encyclopedia of Genes and Genomes (KEGG) enrichment analysis was performed using clusterProfiler (http://www.bioconductor.org/packages/release/Bioc/html/clusterProfiler.html, accessed on 6 June 2022). The transcriptome assembly quality was evaluated through the Benchmarking Universal Single-Copy Orthologs (BUSCO) tool v5.4.3 [49].

### 2.3. Total RNA Extraction and Quantitative Real-Time PCR (qRT-PCR) Analysis

Total RNA of the mycelia of the HN08 strain and Δ*CsAtf1* mutant were extracted using the RNAprep Pure Plant Kit (Tiangen, Beijing, China). cDNA synthesis was performed with TransScript One-Step gDNA Removal and cDNA Synthesis SuperMix (TransGen Biotech, Beijing, China). The expression levels of the candidate target genes were quantified by quantitative real-time PCR (qRT-PCR) performed with an ABI7500 sequence detection system (Applied Biosystems, Waltham, MA, USA). Reactions were performed in a total volume of 10 μL using the SYBR Premix Dimer Eraser Kit (Takara, Beijing, China). All of the reactions were repeated in at least three independent pools in three sets of biological replicates. The primer sequences are listed in Supplementary Table S1.

### 2.4. CsCYP51G1 Gene Cloning and Sequence Analysis

Sequence was obtained by local BLAST search using query sequence (gene ID: 43616755 in Table S2) from whole genome sequence of HN08 strain. Primers CsCyp51G1-OF/CsCyp51G1-OR were designed and listed in Table S1, DNA and cDNA sequence of *CsCYP51G1* were amplified and sequenced. The conserved structural domains were analyzed with Simple Modular Architecture Research Tool (SMART, http://smart.embl-heidelberg.de, accessed on 1 July 2022). Multiple sequences alignment was aligned with clustalW [50]. The alignment was used to construct phylogenetic trees by maximum likelihood method in MEGA6 [51]. The phylogenetic tree was supported with 1000 bootstrap values.

### 2.5. Electrophoretic Mobility Shift Assay (EMSA)

The CsAtf1 protein was expressed using a prokaryotic expression system. The plasmid pET-32a-CsAtf1 was constructed by inserting the *CsAtf1* gene into the vector pET-32a, and the expressed protein contained a 6× His amino acid tag at the N-terminus. The His-CsAtf1 protein was obtained and purified from *Escherichia coli* strain BL21 containing the vector pET-32a-CsAtf1. The DNA probes were amplified by PCR, purified with a Gel Extraction Kit (Tiangen, Beijing, China), and then labelled using the EMSA Probe Biotin Labeling Kit (Beyotime, Shanghai, China) according to the manufacturer’s instructions. An EMSA was performed using the Chemiluminescent EMSA Kit (Beyotime, Shanghai, China). The protein-DNA interaction samples were analysed by 4% nondenaturing polyacrylamide gel electrophoresis in 0.5× TBE buffer. The gel was then transferred to a nylon membrane (Beyotime, Shanghai, China) followed by UV crosslinking.

### 2.6. Yeast One-Hybrid (Y1H) Assays

For the Y1H assay, the promoter of *CsCyp51G1* was inserted between the *Eco*RI and *Sac*I sites in the bait vector pHIS2 [52] to generate pHis2-P-CsCyp51G1. The full-length sequence of CsAtf1 was cloned between the *Bam*HI and *Eco*RI sites of the vector pGADT7 [52] to obtain pGADT7-CsAtf1. Then, the pGADT7-CsAtf1 and pHis2-P-CsCyp51G1 plasmids were introduced into the Y187 yeast strain, and the resultant strain was grown on SD/-Trp-Leu medium with or without His plus 3-AT (80 mM). Cotransformation of the plasmids pGADT7-53 and pHIS2-53 was performed as a positive control, while the pGADT7 and pHIS2 empty vectors were used as negative controls. The plates were then incubated for 3–4 d at 28 °C.

### 2.7. CsCyp51G1 Gene Deletion

**Figure 1:**
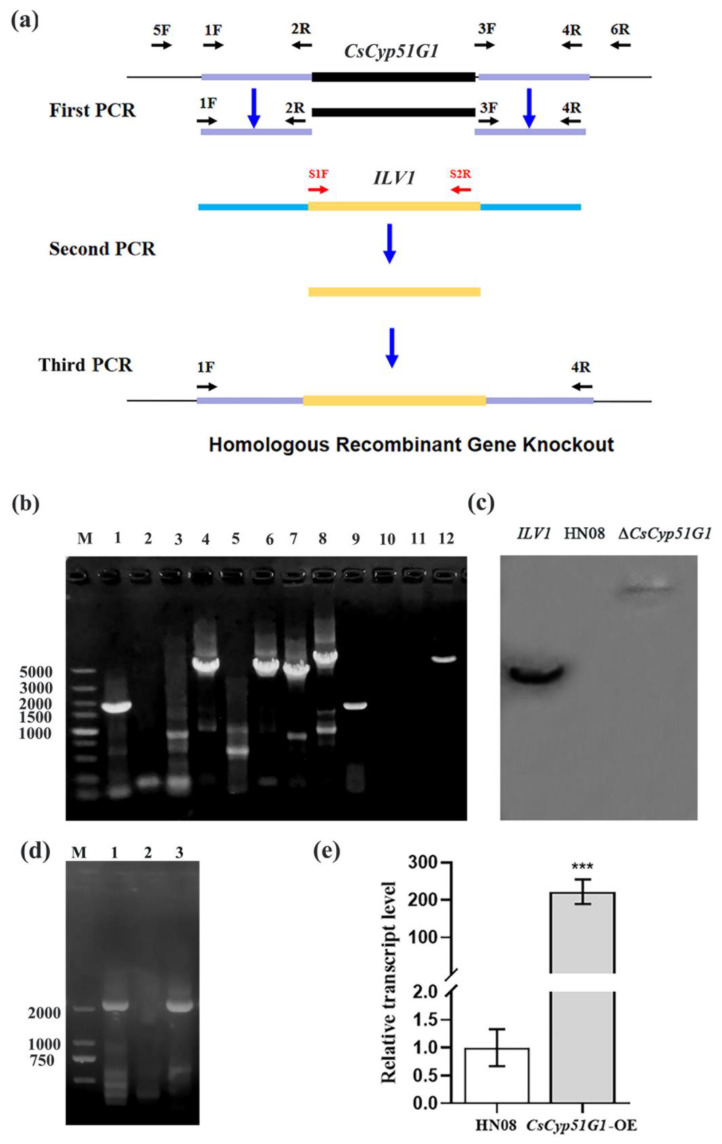
Verification of the gene deletion mutant Δ*CsCyp51G1* and overexpression strain *CsCyp51G1-OE*. (**a**) Schematic diagram of *CsCyp51G1* gene deletion and primers used to verify the gene replacement event. (**b**) Confirmation of the Δ*CsCyp51G1* mutant and *CsCyp51G1-OE* strain by PCR amplification. M: DNA DL 5000 marker; A 1712 bp fragment of the *CsCyp51G1* gene coding sequence was amplified by the primer pair *CsCyp51G1*-OF/OR in both HN08 (lane 1) and *CsCyp51G1-OE* (lane 9) but not Δ*CsCyp51G1* (lane 5). A 4217-bp fragment containing the upstream sequence of the *CsCyp51G1* gene and a partial *ILV1* gene sequence was amplified by the primer pair 5F/S2R from Δ*CsCyp51G1* (lane 6) but not HN08 (lane 2) or *CsCyp51G1-OE* (lane 10). A 4018-bp fragment containing the downstream sequence of the *CsCyp51G1* gene and a partial *ILV1* gene sequence was amplified by the primer pair S1F/6R from Δ*CsCyp51G1* (lane 7) but not HN08 (lane 3) or *CsCyp51G1-OE* (lane 11). A 5267-bp fragment from Δ*CsCyp51G1* (lane 8) and a 4313-bp fragment from HN08 (lane 4) and *CsCyp51G1-OE* (lane 12) were amplified by the primer pair 5F/6R, which confirmed that the 1712-bp fragment of the *CsCyp51G1* gene was replaced by the 2817-bp fragment of the *ILV1* gene. (**c**) Southern blot analysis of total genomic DNA samples from WT and Δ*CsCyp51G1* were digested with *Eco*RI. The probe was an *ILV1* PCR fragment amplified from the sulfonylurea resistance cassette of the plasmid pCX62-S. (**d**) PCR with the primer pair RP27F/CsCyp51G1-OR amplified a 2189-bp fragment from the plasmid pXY203-RP27-Cyp51G1 and the CsCyp51G1-OE overexpression strain but not the wild-type strain. (**e**) Relative expression of *CsCyp51G1* determined by qRT-PCR in *CsCyp51G1-OE* strains and WT HN08. Expression levels were normalized using *ACT* expression levels as controls. Data were collected from three technical replicates. Error bars represent the standard deviations, *** indicated significant differences at *p* < 0.001 (Student’s paired two-tailed *t*-test).-b.

Homologous recombination was performed for *CsCyp51G1* gene deletion. Primer pairs were designed as per the schematic diagram shown in Figure 1a and are listed in Table S1. First, the upstream and downstream flanking sequences of *CsCyp51G1* were amplified with the primer pairs 1F/2R and 3F/4R; 2R and 3F contained the linker sequences of the chlorimuron resistance gene (*ILV1*). Second, the whole *ILV1* gene fragment was amplified by S1F/S2R from the plasmid pcx62-S [28]. Finally, the first- and second-round PCR products were used as templates, and enriched fusion fragments were obtained by fusion PCR amplification using 1F and 4R as primers. The final fusion fragments were directly transformed into protoplasts of HN08, and transformants were screened as described by Song et al. [28].

To determine the number of *ILV1* genes inserted, genomic DNA of Δ*CsCyp51G1* was extracted and digested with *Eco*RI. The genomic DNA of the WT strain HN08 was used as a negative control, and the vector pCB1532 with the *ILV1* gene was used as a positive control. They were hybridized with the 616-bp PCR probe of the partial sequence of the *ILV1* gene amplified with the primers S2F and S1R and labelled with digoxigenin (DIG)-dUTP using the DIG Hing Prime DNA Labeling and Detection Starter Kit I (Roche, Basel, Switzerland).

### 2.8. CsCyp51G1 Gene Overexpression

The overexpression vector pXY203-RP27-Cyp51G1 was constructed by yeast homologous recombination. The pXY203 vector [28] containing the hygromycin transferase gene *HPH* and *RP27* promoter was digested with *Xho*I, the *CsCyp51G1* fragments with homology arms at both ends were obtained by PCR, and both were cotransformed into the yeast strain XK1-25. Clones were screened on SD/-Trp medium. Then, the plasmid pXY203-RP27-Cyp51G1 was extracted from yeast and transformed into *E. coli* for replication. The plasmid pXY203-RP27-Cyp51G1 was transformed into protoplasts of HN08, and transformants were selected on PDS medium (PDA medium with 1 M sucrose added) supplemented with 600 μg/mL hygromycin.

### 2.9. Ergosterol Content Determination

The ergosterol content of the WT HN08, Δ*CsCyp51G1* mutant, *CsCyp51G1*-OE, Δ*CsAtf1* mutant, and Δ*CsPbs2* mutant strains was quantified by high-performance liquid chromatography (HPLC). HPLC analyses were performed using an Agilent 1200 HPLC system. The separation was performed on an Agilent C18 column (250 mm × 4.6 mm, 0.5 μm) with a flow rate of 1 mL/min at 25 °C and eluted with a methanol-0.1% phosphoric acid water (ratio: 60–40) system. The detection wavelength was 281 nm, and the injection volume was 10 μL. The retention time of the peak was 12 min. The standard curves were prepared using 95% pure ergosterol at concentrations ranging from 0.5 ppm to 80 ppm to examine the response (peak area) to different ergosterol concentrations. The data represent the means of three replicates.

### 2.10. Statistical Analysis

All results are expressed as the mean ± standard deviation values. Statistical analysis was performed by ANOVA, followed by Tukey post hoc analyses. Student’s paired *t* test was also used to compare the difference in values between 2 groups. A *p* value < 0.05 was considered statistically significant.

## 3. Results

### 3.1. Identification of Downstream Target Genes Regulated by CsAtf1

To gain insight into the regulatory function of CsAtf1, transcriptome analysis was performed by high-throughput RNA-Seq on the WT and Δ*CsAtf1* mutant strains with three biological replicates. To date, the reference genome and annotations of *C. siamense* have been lacking. For reference-guided analysis, the *C. fructicola* genome was used as the reference assembly in this study [42]. Approximately 44 million paired-end (PE) reads (~6 Gb) from each sample were generated using the BGISEQ-500 platform. The clean reads of the WT and Δ*CsAtf1* mutant were mapped to the reference genome, and the average mapping ratio achieved was 73%. Approximately 75% of the mapped reads were located in exonic regions of the reference genome. Overall, the total number of expressed genes was 13,214. The transcriptome assembly quality was evaluated through the “fungi_odb10” in BUSCO library as reference dataset. The result showed 99.9% completement of a total of 758 BUSCOs.

A total of 4518 genes were significantly differentially expressed in the Δ*CsAtf1* mutant. Among them, 2354 genes and 2164 genes were upregulated and downregulated, respectively. GO enrichment analysis was carried out for the significantly regulated genes (Figures S1 and S2). For genes upregulated in the Δ*CsAtf1* mutant, several functions related to ribosome biogenesis, RNA processing, and binding function were significantly enriched. Among them, ribosome biogenesis (*p*-value: 9.5 × 10^−34^) was the most significantly enriched process. The largest gene set had functions related to binding, e.g., ribonucleotide binding (*p*-value: 2.9 × 10^−10^), ATP binding (*p*-value: 2.2 × 10^−10^), and drug binding (*p*-value: 1.1 × 10^−9^). Functions significantly enriched by downregulated genes differed from those enriched by upregulated genes. The most significantly enriched biological process was related to metabolic processes, e.g., cofactor metabolic processes (*p*-value: 1.2 × 10^−11^), small molecule metabolic processes (*p*-value: 1.9 × 10^−10^), and drug metabolic processes (*p*-value: 4.0 × 10^−9^).

Functional classification of Kyoto Encyclopedia of Genes and Genomes (KEGG) pathways was also carried out (Figures S3 and S4). Genes within the same KEGG pathway usually cooperate to perform biological functions. There was a notable difference in KEGG pathways between upregulated and downregulated genes. Among upregulated genes, four KEGG pathways were significantly enriched, i.e., “Ribosome biogenesis in eukaryotes” (*p*-value: 5.2 × 10^−12^), “MAPK signaling pathway-yeast” (*p*-value: 3.8 × 10^−6^), “RNA polymerase” (*p*-value: 6.3 × 10^−6^), and “RNA transport” (*p*-value: 0.03). At the same time, 13 KEGG pathways were enriched by downregulated genes. Most of these biological processes were related to biosynthesis and metabolism, e.g., “Biosynthesis of antibiotics” (*p*-value: 2.0 × 10^4^), “Biosynthesis of amino acids” (*p*-value: 3.9 × 10^−4^), “Carbon metabolism” (*p*-value: 8.3 × 10^−4^), and “Sulfur metabolism” (*p*-value: 0.025).

### 3.2. Genome-Scale Exploration of CsAtf1 Binding Loci

To investigate the molecular mechanisms underlying the pleiotropic phenotypes caused by *CsAtf1* deletion, we performed a ChIP assay to isolate the Atf1-binding DNA fragment and then analysed it by next-generation sequencing (ChIP-seq). Approximately 42.8 million PE reads (~6.3 Gb) were obtained in each sample from ChIP-Seq experiments and aligned to the reference genome sequence. The genomic loci bound by CsAtf1 were recognized as high-density aligned areas or peaks. A total of 30,045 statistically significant peaks with an average length of 488 bp were called.

The genomic distribution of peaks with regard to gene structure was investigated (Figure S5). A total of 48.23% of the total peaks were located in gene promoters, whereas others were bound in gene exons (35.78%), introns (4.93%), and intergenic regions (11.06%). A total of 12,730 genes were predicted as being recognized by CsAtf1 and could be classified into different categories by GO and KEGG analyses (Figures S6 and S7). The most significantly enriched cellular component was the “endomembrane system” (*p*-value: 7.7 × 10^−6^), a collection of membranous structures involved in transport within cells, especially the “endoplasmic reticulum” (*p*-value: 2.1 × 10^−4^). Motif analysis for peaks was performed using HOMER [53], with de novo motif discovery and known motif screening. The top three significantly enriched motifs were “CTTCCGTCTACG”, “GTACAGCTTATG”, and “ATCCGTTCTGAC”. The motif with the most significant *p*-value was “CTTCCGTCTACG” (*p*-value: 1.0 × 10^−43^) (Figure S8). All three motifs had no matches to known motifs.

### 3.3. Integrative Analysis of RNA-Seq and ChIP-Seq Data Showed That Some Cytochrome Oxidase Genes Were Candidate CsAtf1 Target Genes

**Figure 2:**
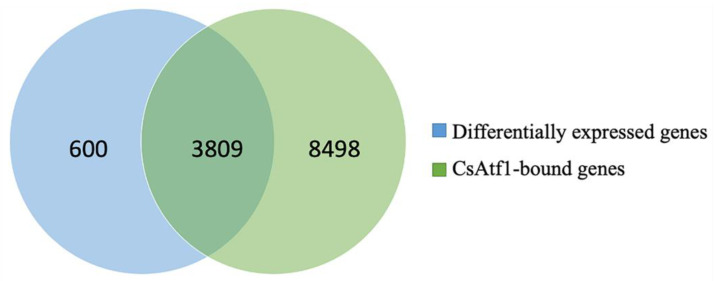
Venn diagram of significantly differentially expressed genes in the Δ*CsAtf1* mutant and genes bound directly by the transcription factor CsAtf1.

The RNA-Seq and ChIP-Seq analyses were integrated to examine the genome-wide distribution patterns of CsAtf1-binding loci and their relationships with gene expression. A total of 3809 significantly differentially expressed genes were predicted as being directly regulated by CsAtf1 (Figure 2), among which 2029 and 1780 genes were up- and downregulated in the Δ*CsAtf1* mutant, respectively. A total of 5922 peaks were associated with the regulated genes in the ChIP-Seq data. They predominantly localized to exonic (~49%) and upstream (~44%) regions, indicating that CsAtf1 directly targeted and regulated a set of genes by binding to exonic and promoter regions. Notably, 24 cytochrome oxidase genes were predicted as being targeted directly by CsAtf1 (Table S2). Cytochrome oxidases are the primary targets of numerous fungicides [29,30]. Therefore, the targeted cytochrome oxidase genes identified in this study may contribute to the understanding of the fungicide sensitivity of CsAtf1.

### 3.4. CsAtf1 Acts as an Upstream Regulator to Negatively Regulate the Expression of CsCyp51G1

**Figure 3:**
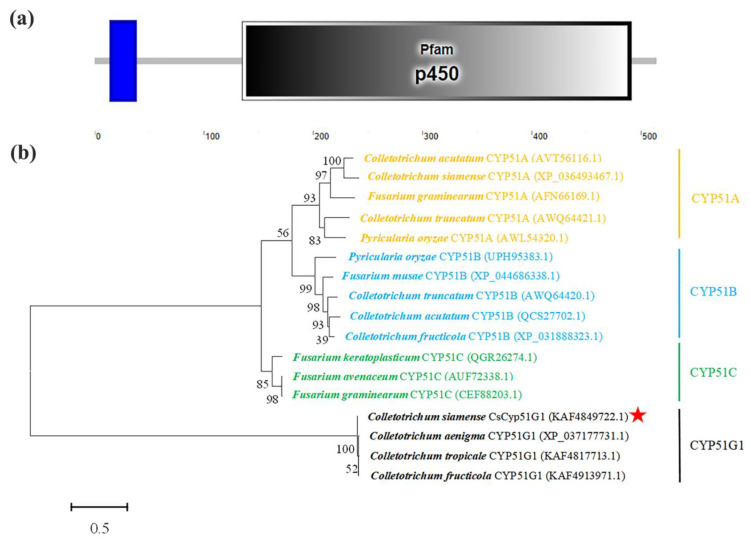
Protein domains and phylogenetic analysis of CsCyp51G1. (**a**) SMART analysis of the CsCyp51G1 protein domain. (**b**) Phylogenetic analysis of CsCyp51G1 and its homologues from other fungal species. The phylogenetic tree was constructed with MEGA 6.0 using the maximum likelihood method. The CsCyp51G1 protein amino acid sequence in this study is indicated with a red star.

Sterol 14 alpha-demethylase (*CYP51*) is the most widely distributed member of the CYP gene superfamily, and it is a well-known drug target for treating microbial pathogenic infections [54]. In this study, we observed that a *CYP51*-encoding gene (*CYP51G1*, Gene ID: 43616755) was a candidate target gene of CsAtf1 (Table S2). Hence, we first confirmed the relationship between the *CYP51G1* gene and CsAtf1. The DNA and cDNA of *CYP51G1* were amplified from the *C. siamense* HN08 strain. Sequence analysis showed that it had a DNA size of 1712 bp and a cDNA size of 1548 bp, encoding a 514 amino acid peptide with a conserved P450 structural domain (oxidation-reduction process; *E* value: 3 × 10^−24^). Phylogenetic analysis showed that the amplified gene was *Cyp51G1* rather than *CYP51A*, *CYP51B,* or *CYP51C* (Figure 3). We named it *CsCyp51G1* and deposited the sequence into GenBank (accession no. OP122501).

**Figure 4:**
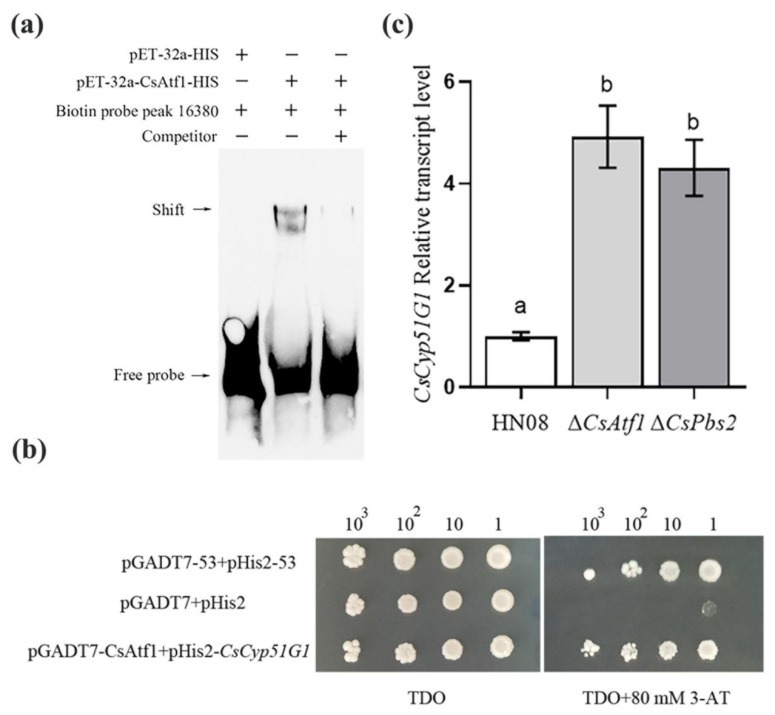
Analysis of the binding and expression relationship between CsAtf1 and *CsCyp51G1*. (**a**) Detection of the binding relationship between the CsAtf1 protein and the promoter regions of *CsCyp51G1* by EMSA. +, protein added, −, protein not added. (**b**) Y1H Gold confirmed that the transcription factor CsAtf1 binds to the promoter of *CsCyp51G1*. The representative growth status of different cell culture dilutions (1:1, 1:10, 1:100, 1:1000) on SD/-Leu/-Trp/-His agar medium without and with 80 mM 3-AT is shown. (**c**) Relative expression analysis of *CsCyp51G1* in the wild-type HN08, Δ*CsPbs2*, and Δ*CsAtf1* strains. Different letters (a, b) represent significant difference at *p* < 0.01 (One-way ANOVA and Duncan’s test).

The transcription factor CsAtf1 exhibited binding to the promoter region (peak_16380) of *CsCyp51G1* in the ChIP-Seq data, which was experimentally verified by an EMSA and a Y1H assay in this study. In the EMSA (Figure 4a), a 273-bp DNA fragment of the *CsCyp51G1* promoter containing peak_16380 was amplified, and its binding relationship with the CsAtf1 protein was verified. The results indicated that the *CsCyp51G1* promoter and CsAtf1 protein formed stable protein-DNA complexes, as indicated by the slow-retardation band present in the experimental group (lane 2) but not in the control group (lane 1), and the cold-competition group with an unlabeled probe showed attenuated intensity of the supershifted band (lane 3). This experiment confirmed that the CsAtf1 protein could bind directly to the promoter of *CsCyp51G1* in vitro. In the Y1H assay (Figure 4b), both the vector pGADT7-CsAtf1 with the full-length cDNA of *CsAtf1* and pHis2-P-CsCyp51G1 with a 1500-bp *CsCyp51G1* promoter were constructed and introduced into the Y187 yeast strain. All the yeast colonies grew well on selective dropout medium (SD/-Leu/-Trp/-His) without 3-amino-1,2,4-triazole (3-AT). However, on medium with 80 mM 3-AT, the experimental group (pGADT7-CsAtf1 and pHis2-P-CsCyp51G1) and positive control (pGADT7-53 and pHIS2-53) grew normally, while the negative control (pGADT7 and pHIS2 empty vector) could not survive. The binding interaction between the CsAtf1 protein and the *CsCyp51G1* promoter region was also evident. Therefore, by combining ChIP-Seq, EMSA, and an Y1H assay, the transcription factor CsAtf1 was confirmed to interact directly with the *CsCyp51G1* promoter.

RNA-Seq data showed that the *CsCyp51G1* gene was upregulated in the Δ*CsAtf1* mutant compared with the WT. The transcriptional expression levels of *CsAtf1* and *CsCyp51G1* were evaluated using qRT-PCR in the Δ*CsAtf1* mutant, Δ*CsPbs2* mutant, and WT strain HN08 (Figure 4c). The results showed that the transcription levels of *CsCyp51G1* were significantly upregulated in the Δ*CsAtf1* and Δ*CsPbs2* mutants compared with the WT. The measured expression levels of *CsCyp51G1* in the Δ*CsAtf1* mutant and in the Δ*CsPbs2* mutant were approximately 4.92- and 4.31-fold higher than that in the WT, respectively. Therefore, the qRT-PCR results further confirmed the results of the RNA-Seq analyses. These results indicated that the transcription factor CsAtf1 and the upstream gene *CsPbs2* negatively regulated the expression of *CsCyp51G1*. Combined with the binding relationship and transcriptional expression assay, the results of the study illustrated that CsAtf1 acts as an upstream regulator to negatively regulate the expression of *CsCyp51G1*.

### 3.5. CsCyp51G1 Was Involved in Fludioxonil Sensitivity Regulation in C. siamense

Our previous studies showed that the transcription factor CsAtf1 is involved in the regulation of fludioxonil sensitivity in *C. siamense* [28]. Since *CsCyp51G1* is the target gene of CsAtf1, we further evaluated whether *CsCyp51G1* is involved in fludioxonil sensitivity regulation in *C. siamense*.

The *CsCyp51G1* gene deletion mutant was generated by targeted replacement of the 1712-bp *CsCyp51G1* gene fragment with the 2817-bp *ILV1* gene fragment (Figure 1a). A Δ*CsCyp51G1* mutant from nine fungal transformants was confirmed as a null mutant by PCR amplification, sequencing, and Southern blot analysis. The internal sequence of the *CsCyp51G1* gene (Figure 1b, lane 5) could not be amplified by the primers Cyp51F/R, and the bands in lanes 6 and 7 amplified by the primers 5F/S2R and S1F/6R, respectively, appeared at the predicted sizes in the mutant Δ*CsCyp51G1*, which indicated that the *ILV1* gene replaced *CsCyp51G1*. Then, the PCR products amplified from Δ*CsCyp51G1* and HN08 genomic DNA using the primers 5F/6R were sequenced, yielding a 5267-bp fragment from Δ*CsCyp51G1* and a 4313-bp fragment from HN08. This indicated that the 1712-bp open reading frame of the *CsCyp51G1* gene was exactly replaced by the 2817-bp fragment of the *ILV1* gene, as shown in Figure 1a. Southern blot analysis showed that the Δ*CsCyp51G1* mutant contained only one copy of the *ILV1* gene (Figure 1c). Therefore, Δ*CsCyp51G1* was indeed a *CsCyp51G1* null mutant.

The overexpression plasmid pXY203-RP27-Cyp51G1 was also constructed, for which the vector backbone was pXY203 with the hygromycin transferase encoding gene *HPH*, and the open reading frame of *CsCyp51G1* fused with a strong RP27 promoter. Then, transformants of *CsCyp51G1-OE* were obtained by integrating the plasmid pXY203-RP27-Cyp51G1 into the genome of the WT strain HN08 by PEG-mediated transformation under selective media, and the construct was confirmed by PCR and sequenced. The results showed that the RP27 promoter and *CsCyp51G1* coding sequence could be amplified by the primer RP27-F from the promoter RP27 and primer CsCyp51G1-OR from the *CsCyp51G1* gene open reading frame in the overexpression strain *CsCyp51G1-OE* (Figure 1d, lane 3) and from the positive control plasmid pXY203-RP27-Cyp51G1 (Figure 1d, lane 1) but not from the negative control WT strain (Figure 1d, lane 2). The band in lane 3 was confirmed by sequencing, RP27-CsCyp51G1 was introduced into the genome of the WT strain, and the *CsCyp51G1-OE* strain was constructed. The expression of *CsCyp51G1* was measured by qRT-PCR analysis to determine whether *CsCyp51G1* was overexpressed in the *CsCyp51G1-OE* strain. The results showed that the *CsCyp51G1* expression level in *CsCyp51G1-OE* was 220-fold higher than that in the WT (Figure 1e), which confirmed that *CsCyp51G1-OE* was a *CsCyp51G1* overexpression strain.

**Figure 5:**
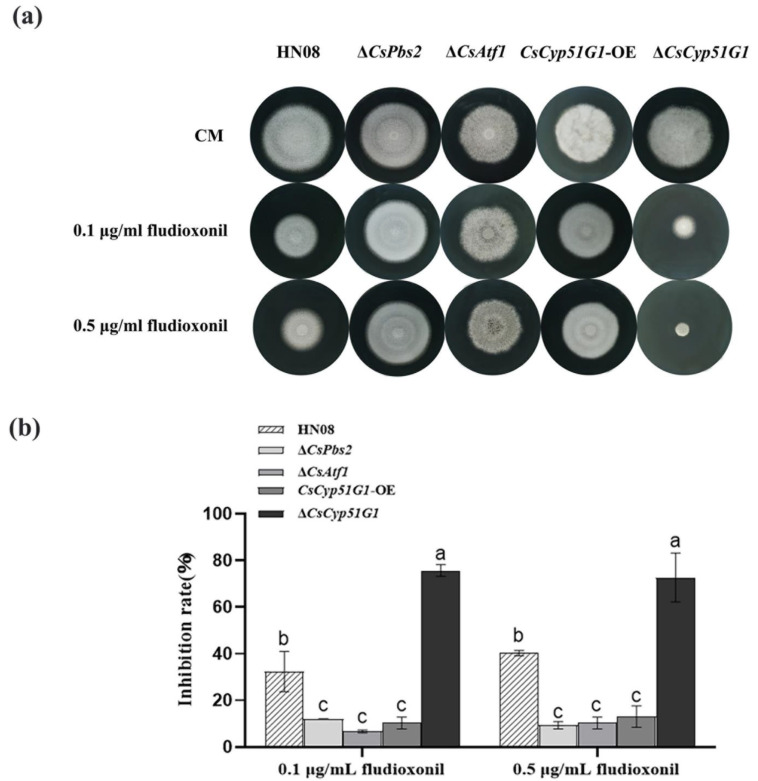
Comparison of fludioxonil resistance among the WT strain HN08, Δ*CsPbs2*, Δ*CsAtf1*, *CsCyp51G1-OE,* and Δ*CsCyp51G1*. (**a**) Mycelial growth of the tested strains cultured in CM containing different concentrations of fludioxonil for 5 d. (**b**) Growth inhibition rate of the tested strains under different concentrations of fludioxonil. The growth inhibition rate is relative to the growth rate of each untreated control [(diameter of untreated strain − diameter of treated strain)/(diameter of untreated strain × 100%)]. Three repeats were performed. The error bars show the SD value, and different letters indicate significant difference at *p* < 0.01 (One-way ANOVA and Duncan’s test).

The mycelial growth of the WT HN08, Δ*CsCyp51G1* mutant, *CsCyp51G1-OE* strain, Δ*CsAtf1* mutant, and Δ*CsPbs2* mutant strains on CM plates with fludioxonil was compared (Figure 5). The results showed that there were no significant differences in colony morphology or conidial size in CM. However, under fludioxonil stress conditions, the mycelial growth rate of the *CsCyp51G1*-OE strain was significantly higher than that of the WT, similar to that of the Δ*CsPbs2* deletion and Δ*CsAtf1* mutant strains, while the colony size of the Δ*CsCyp51G1* mutant exhibited a dramatic reduction (Figure 5). This result suggested that overexpression of the *CsCyp51G1* gene can increase fludioxonil resistance and that absence of *CsCyp51G1* expression can increase the fludioxonil sensitivity of *C. siamense*. These results revealed that the *CsCyp51G1* gene is involved in fludioxonil sensitivity regulation and that the transcription factor CsAtf1 negatively regulates the *CsCyp51G1* gene, leading to increased fludioxonil tolerance in *C.* *siamense*.

**Figure 6:**
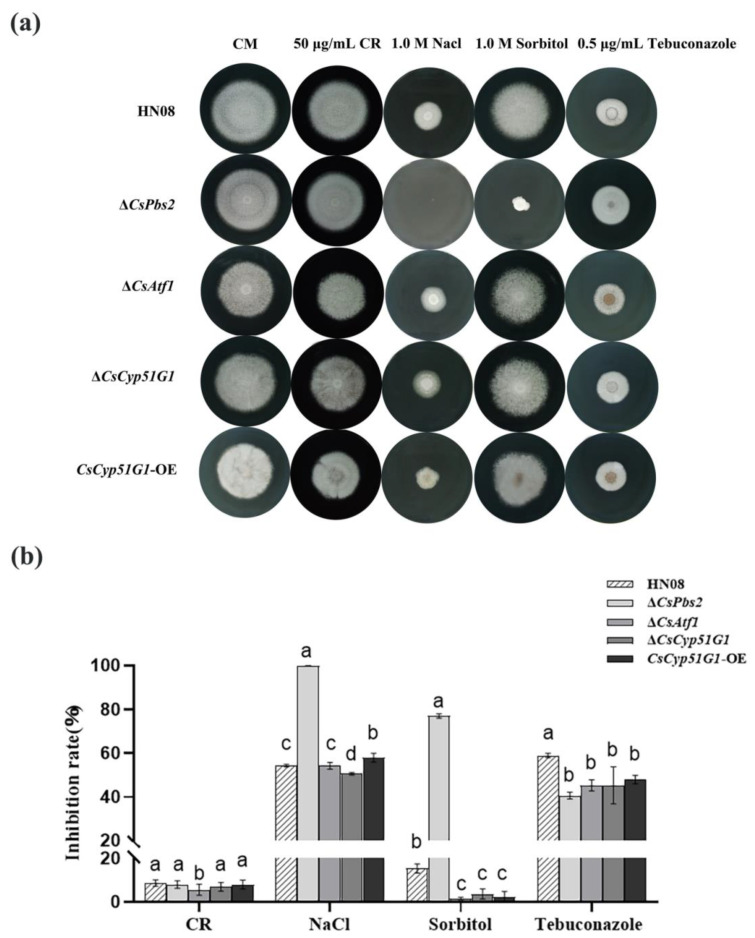
Comparison of responses to various stresses among HN08, Δ*CsPbs2*, Δ*CsAtf1*, *CsCyp51G1-OE,* and Δ*CsCyp51G1*. (**a**) Mycelial growth of HN08, Δ*CsPbs2*, Δ*CsAtf1*, *CsCyp51G1-OE,* and Δ*CsCyp51G1* on CM containing 1 M NaCl, 1 M sorbitol, 50 μg/mL Congo red, and 0.5 μg/mL tebuconazole for 5 d. Approximately 1 × 10^6^ conidia of each strain were inoculated onto the center of the CM plates and grown for 5 d. (**b**) Growth inhibition rates of the tested strains under different stresses. The growth inhibition rate is relative to the growth rate of each untreated control [(diameter of untreated strain − diameter of treated strain)/(diameter of untreated strain × 100%)]. Three repeats were performed. Error bars represent the standard deviations. Different letters (a, b, c, d) represent significant difference at *p* < 0.01 (One-way ANOVA and Duncan’s test).

In addition, we investigated whether *CsCyp51G1* was involved in osmoregulation or virulence. Similar to the Δ*CsAtf1* deletion strains [28], the growth inhibition of Δ*CsCyp51G1* exposed to 1 M NaCl, 1 M sorbitol, and 0.5 μg/mL tebuconazole was slightly lower than that of the WT strain under the corresponding conditions, but there was no significant difference upon exposure to 50 μg/mL Congo Red (Figure 6). However, unlike *CsAtf1*, which is involved in virulence, no significant differences in virulence were observed in *CsCyp51G1* mutants compared to the WT (data not shown).

### 3.6. CsCyp51G1 Regulated Fludioxonil Sensitivity but Did Not Affect the Ergosterol Content in C. siamense

**Figure 7:**
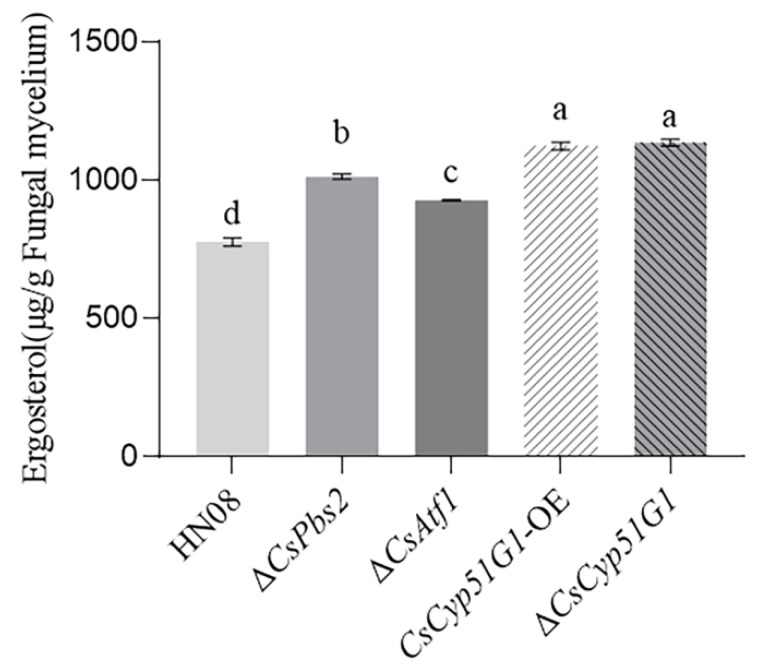
Ergosterol content in HN08, Δ*CsPbs2*, Δ*CsAtf1*, *CsCyp51G1-OE,* and Δ*CsCyp51G1*. The error bars show the SD values, and different letters (a, b, c, d) represent significant difference at *p* < 0.01 (One-way ANOVA and Duncan’s test).

CYP51 is a key enzyme involved in ergosterol biosynthesis in fungi [55]. Ergosterol is a fundamental component of the fungal membrane, and DMI fungicides act by inhibiting ergosterol synthesis, leading to disruption of cell membrane function [56]. We tried to determine whether *CsCyp51G1* affected fludioxonil sensitivity by changing ergosterol levels. The ergosterol content of the WT HN08, Δ*CsCyp51G1* mutant, *CsCyp51G1-OE*, Δ*CsAtf1* mutant, and Δ*CsPbs2* mutant strains was measured using an HPLC assay (Figure 7). The results showed that the cellular ergosterol levels in the Δ*CsPbs2* mutant, Δ*CsAtf1* mutant, and *CsCyp51G1-OE* strains were significantly higher than those in WT HN08. The cellular ergosterol content in the Δ*CsCyp51G1* mutant was also higher than that in the WT. Given that the phenotype of fludioxonil sensitivity of the Δ*CsCyp51G1* mutant was different from that of other strains, we hypothesized that *CsCyp51G1* regulates fludioxonil sensitivity by affecting factors other than the ergosterol content in *C. siamense*.

## 4. Discussion

Our previous studies reported that the HOG MAPK pathway and its downstream transcription factor CsAtf1 are both involved in regulating the sensitivity to fludioxonil [8,28]. In this study, we searched for potential target genes by integrating RNA-Seq and ChIP-Seq. We verified that *CsCyp51G1* is an important downstream target gene of CsAtf1 and that its expression is negatively regulated by CsAtf1. Then, we investigated the function of *CsCyp51G1*, and the results showed that *CsCyp51G1* is involved in fludioxonil sensitivity regulation in *C. siamense*. Furthermore, our study suggested that the fludioxonil sensitivity of *CsCyp51G1* is independently associated with the ergosterol content in *C. siamense*.

Identifying target genes of transcription factors is particularly important since transcription factors function primarily through their interactions with various genes and participate in different signaling pathways [57]. Due to the diverse functions of transcription factors in different species, the current study of the transcription factor Atf1 primarily focused on its function. There have been few studies demonstrating that target genes of Atf1, such as ATF-1 in *Neurospora crassa*, can regulate the expression of the *ccg-1* (clock-controlled gene) and *cat-1* (catalase gene) genes by binding to their promoter via the CRE motif (5′-TGACGTCA-3′). Based on the characterization of the function of CsAtf1 in fludioxonil sensitivity and virulence in *C. siamense* [28], we identified 3809 potential target genes by comprehensive analysis of ChIP-Seq and RNA-Seq data, including some genes known to be involved in fungicide sensitivity and pathogenicity (data not shown). These candidate target genes provide a basis for further elucidation of how CsAtf1 regulates fungicide sensitivity and pathogenicity.

In this study, we focused on the regulatory mechanism of CsAtf1 in fungicide sensitivity, and we focused on a member of the CYP51 subfamily, the gene *CsCyp51G1*, which encodes sterol 14-alpha-demethylase, an enzyme that is essential for the ergosterol synthesis pathway [58]. Several key enzymes in the ergosterol synthetic pathway are potential targets in pathogenic fungi for the development of antifungal drugs [54]. Inhibitors inhibited the activity of these enzymes and blocked the synthesis of ergosterol, which may disrupt the structure of the cell membrane and prevent infection and reproduction. CYP51 is an important enzyme in the biosynthetic process of ergosterol, and DMIs target CYP51 enzymes [59], e.g., *CgCYP51A* and *CgCYP51B* in *C. gloeosporioides* [60]. Here, we characterized the role of *CsCyp51G1* in regulating the sensitivity of *C.* *siamense* to fludioxonil but not azoles.

Regarding the fludioxonil reaction in fungi, evidence has shown that the His–Asp phosphorelay system and HOG MAPK cascade are involved in transcriptional responses to fludioxonil [22]. Studies have shown that the target of fludioxonil is a type III histidine kinase; this compound inhibits phosphorylation by the type III histidine kinase and activates the HOG MAPK pathway [15,61]. The specific downstream mechanism of action of fludioxonil has yet to be identified. Atf1 is a downstream transcription factor of the HOG MAPK pathway, and some researchers have speculated and tried to determine whether Atf1 or target genes of Atf1 are involved in regulating fludioxonil sensitivity. Hagiwara et al. performed genome-wide expression analyses and showed frequent overlaps and expression clustering profiles between genes upregulated or downregulated by fludioxonil in a HogA- and AtfA-dependent manner, which indicated the relationship between HogA and AtfA in fludioxonil sensitivity regulation. However, Hagiwara et al. showed the sensitivity of the Δ*atf-1* strain to fludioxonil, in contrast to the Δos-2 strain. The authors suggested that the growth inhibition effect of fludioxonil was dependent on factors other than AtfA [22]. In *C. siamense*, our group confirmed that *CsPbs2*, the key member of the HOG MAPK signaling pathway, and its downstream transcription factor CsAtf1 are involved in regulating fludioxonil sensitivity [8,28]. Here, we further demonstrated that CsAtf1 negatively regulates the CYP gene *CsCyp51G1* to increase fludioxonil sensitivity. In addition, the other candidate cytochrome oxidase-related genes directly targeted by CsAtf1 are listed in Table S2. We will continue searching for other P450 genes in *C. siamense* to establish the relationship between the HOG MAPK pathway protein CsAtf1 and the growth inhibition effect of fludioxonil.

Studies have shown that the expression levels of HOG pathway members and CYP51s can affect the cellular ergosterol content. For example, in *Cryptococcus neoformans*, the Δ*hog1* (MAPK) and Δ*ssk1* (MAPKKK) mutants were found to generate significantly higher levels of cellular ergosterol than the WT [62], Δ*Erg11B* of *A. fumigatus* exhibited significantly reduced ergosterol content [63], and overexpression of *Erg11* in *Candida albicans* elevated the ergosterol content [64]. Our studies showed that the HOG pathway and the transcription factors CsAtf1 and CsCyp51G1 are involved in fludioxonil sensitivity regulation in *C. siamense*, which prompted us to suggest a relevance between fludioxonil sensitivity and ergosterol biosynthesis. We found that the ergosterol content was significantly higher in the Δ*CsPbs2* and Δ*CsAtf1* mutants than in the WT strain. However, the ergosterol content in both the Δ*CsCyp51G1* and *CsCyp51G1-OE* strains was significantly higher than that in the WT, which was not consistent with their fludioxonil sensitivity phenotype. Therefore, we suggest that HOG MAPK-CsAtf1-CsCyp51G1 does not directly affect fludioxonil sensitivity by affecting the ergosterol content.

**Figure 8:**
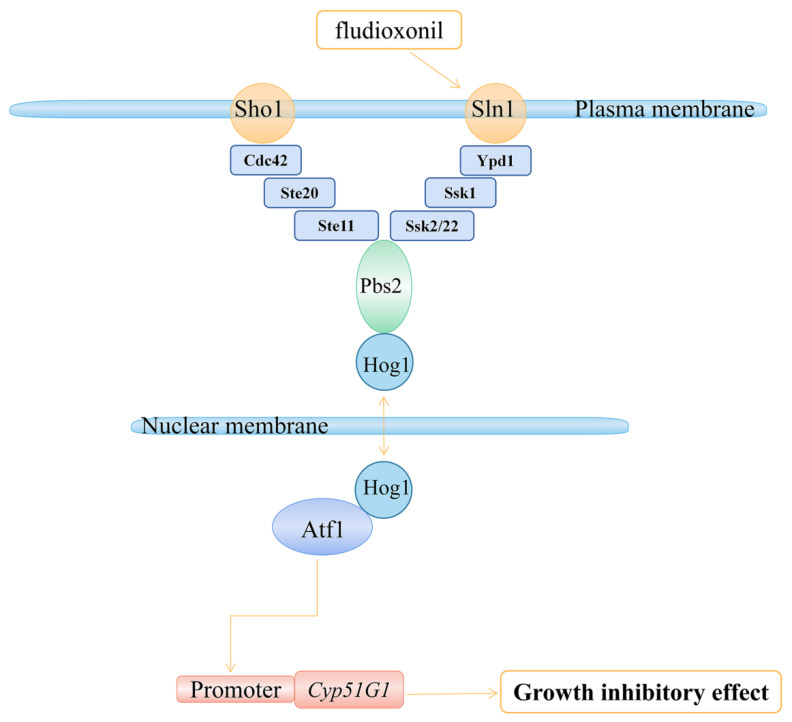
Proposed model from our study showing that CsAtf1 downregulates *CsCyp51G1* expression levels, leading to fludioxonil sensitivity.

In conclusion, our study further revealed the mechanism of action of *C. siamense* in the response to fludioxonil. The transcription factor CsAtf1 can bind to the *CsCyp51G1* promoter and negatively regulate its expression. The *CsCyp51G1* gene is involved in fludioxonil sensitivity regulation in *C. siamense*, which is consistent with our result for Δ*CsAtf1*. Hence, we propose a working model in which CsAtf1 regulates *CsCyp51G1* expression, resulting in increased tolerance to fludioxonil (Figure 8). Our study not only enriched the understanding of the mechanism of HOG MAPK-CsAtf1 but also further revealed the biological mechanism of fludioxonil.

## Data Availability

The data that support the findings of this study are available from the corresponding author on reasonable request.

## Supplementary Materials

The following supporting information can be downloaded at: https://www.mdpi.com/article/10.3390/jof8101032/s1, Figure S1: Gene Ontology enrichment terms of significantly up-regulated genes in Δ*CsAtf1* mutant. Figure S2: Gene Ontology enrichment terms of significantly down-regulated genes in Δ*CsAtf1* mutant. Figure S3: KEGG enrichment pathways of significantly up-regulated genes in Δ*CsAtf1* mutant. Figure S4: KEGG enrichment pathways of significantly down-regulated genes in Δ*CsAtf1* mutant. Figure S5: Venn pie chart of peak distribution of CsAtf1 DNA binding sites in the genome. Figure S6: GO enrichment analyses of genes probably affected by CsAtf1. Figure S7: KEGG enrichment analyses of genes probably affected by CsAtf1. Figure S8: Sequence logos showing the three most enriched motifs in ChIP-Seq. The size of the base represented the possibility of the base appearing. Table S1: List of primers used in the study. Table S2: Cytochrome oxidase related genes that probably directly regulated by CsAtf1.
