# Supplementary material for: The Transcription Factor CsAtf1 Negatively Regulates the Cytochrome P450 Gene CsCyp51G1 to Increase Fludioxonil Sensitivity in Colletotrichum siamense
Source: J Fungi (Basel). 2022 Sep 29;8(10):1032. doi: 10.3390/jof8101032 (PMC9605597; doi:10.3390/jof8101032)
Supplement: Supplementary file 1 [file jof-08-01032-s001.zip › jof-1938102-supplementary.pdf]

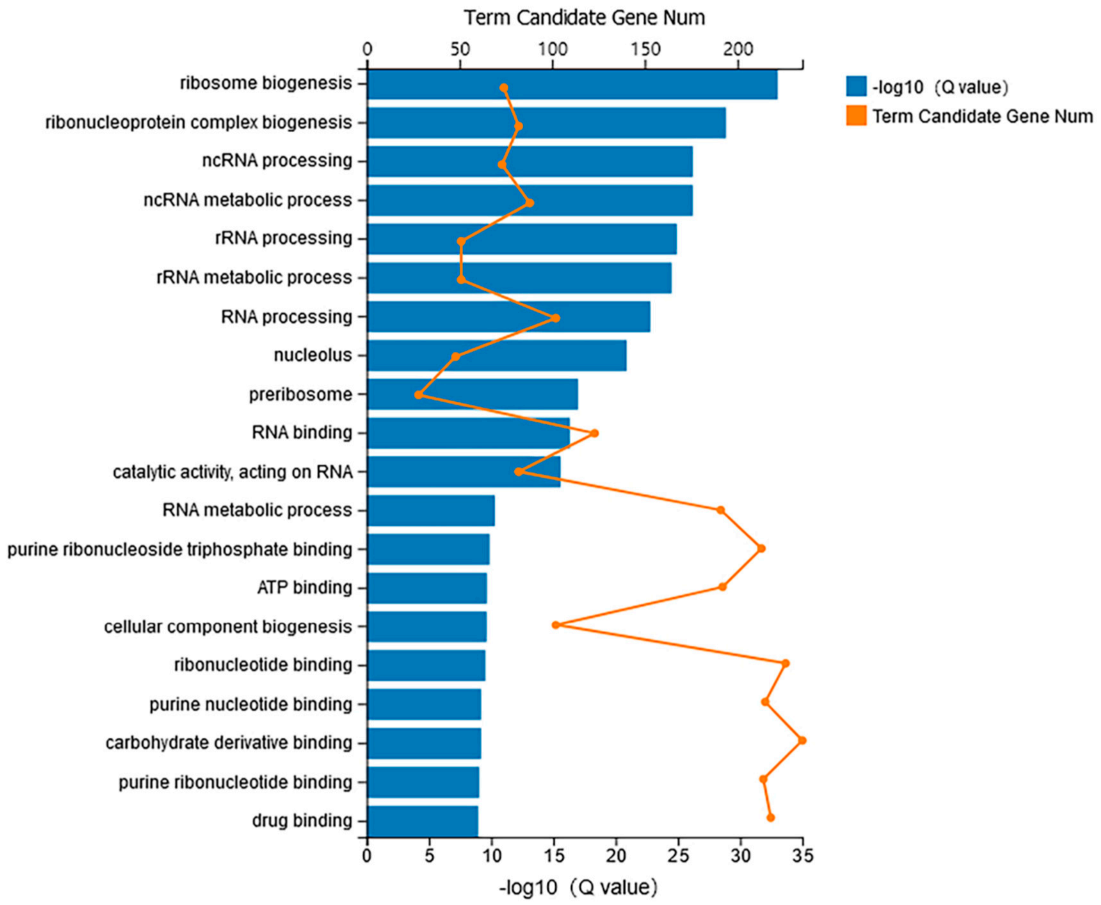

Figure S1. Gene Ontology enrichment terms of significantly up-regulated genes in Δ*CsAtf1* mutant.

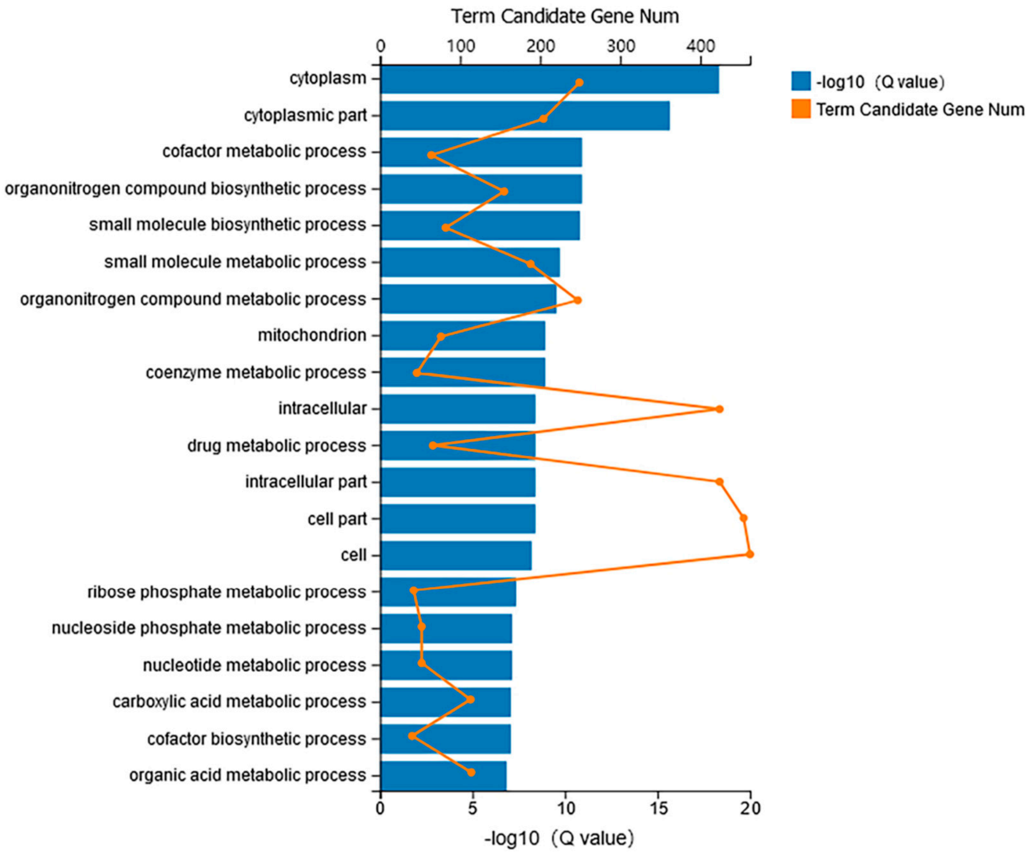

Figure S2. Gene Ontology enrichment terms of significantly down-regulated genes in Δ*CsAtf1* mutant.

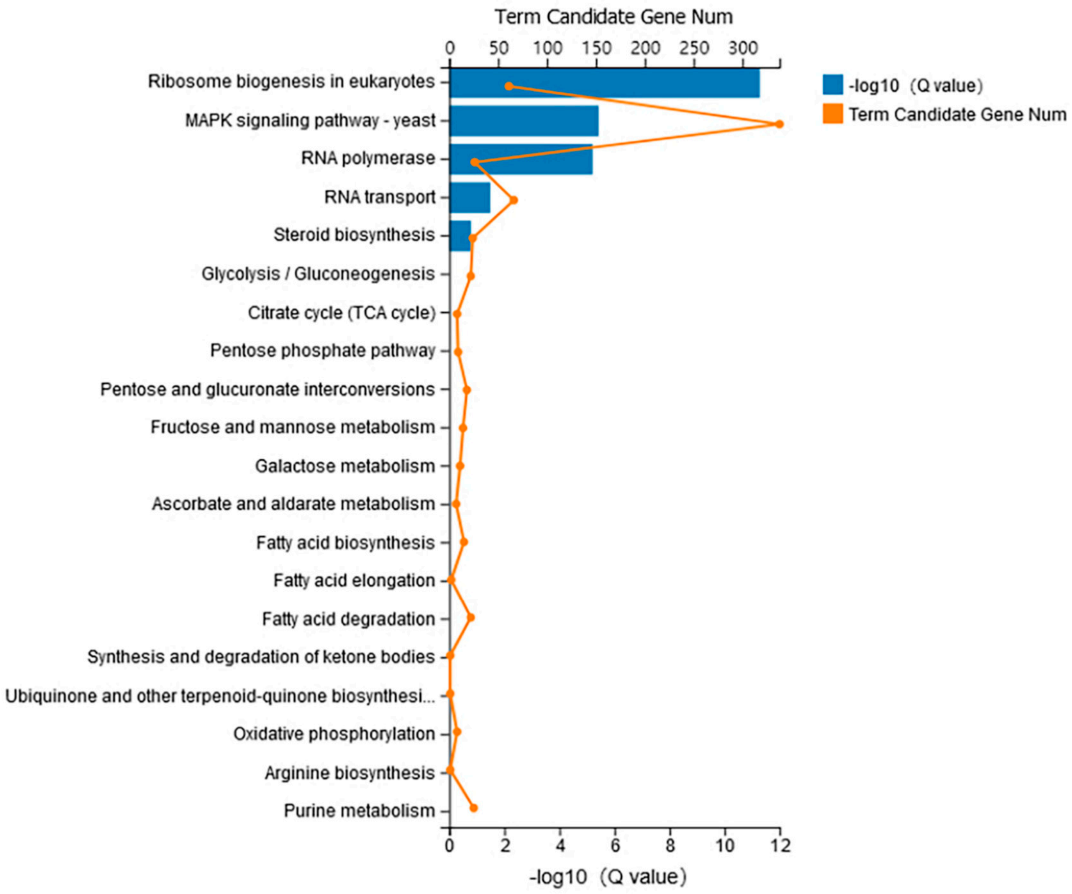

Figure S3. KEGG enrichment pathways of significantly up-regulated genes in Δ*CsAtf1* mutant.

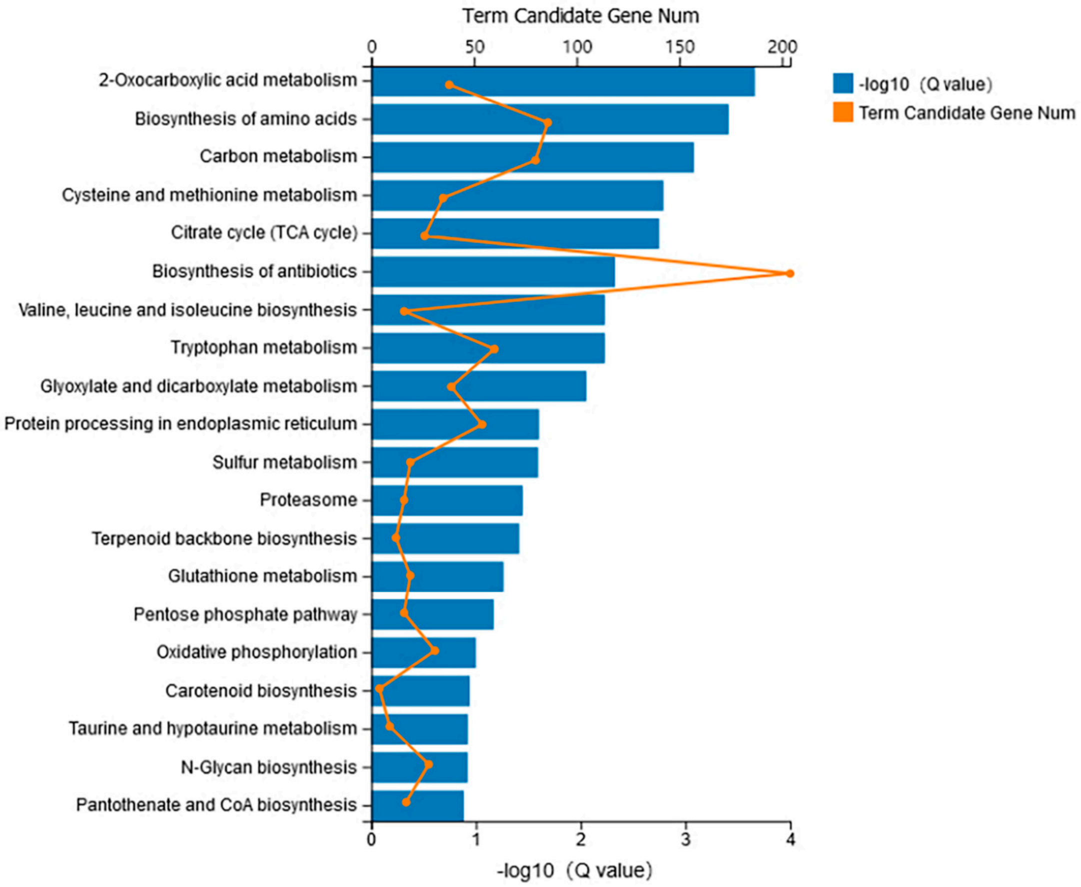

Figure S4. KEGG enrichment pathways of significantly down-regulated genes in Δ*CsAtf1* mutant.

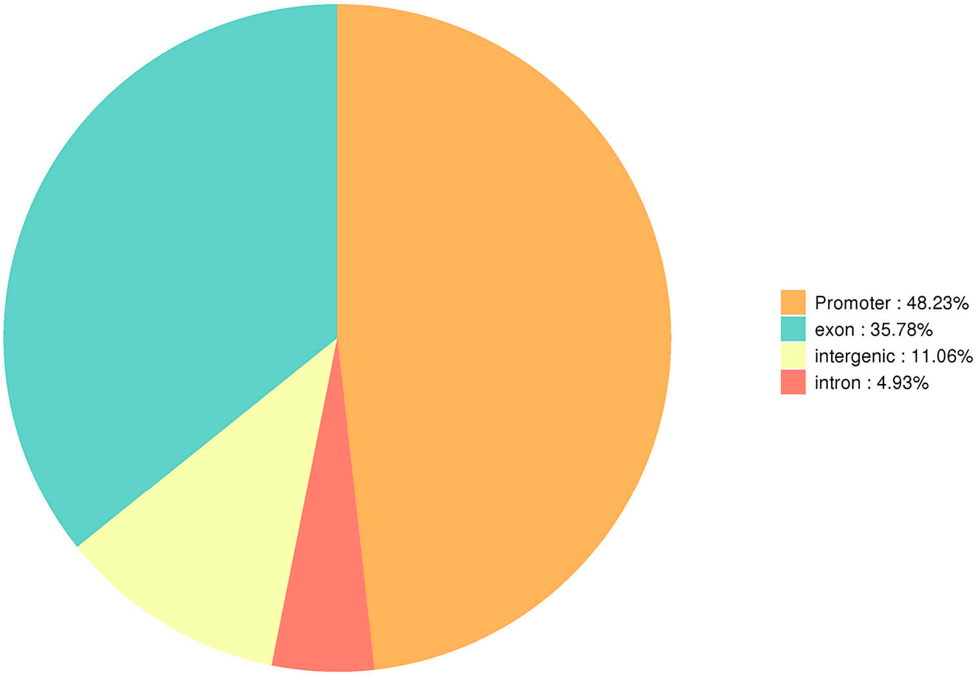

Figure S5. Venn pie chart of peak distribution of CsAtf1 DNA binding sites in the genome.

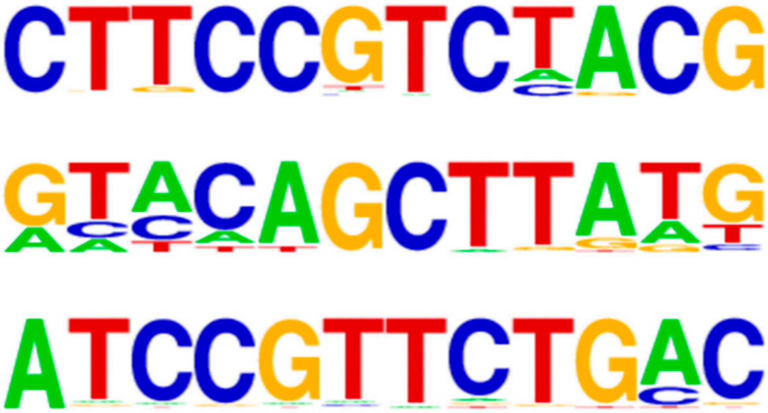

Figure S6. Sequence logos showing the three most enriched motifs in ChIP-seq. The size of the base represented the possibility of the base appearing.

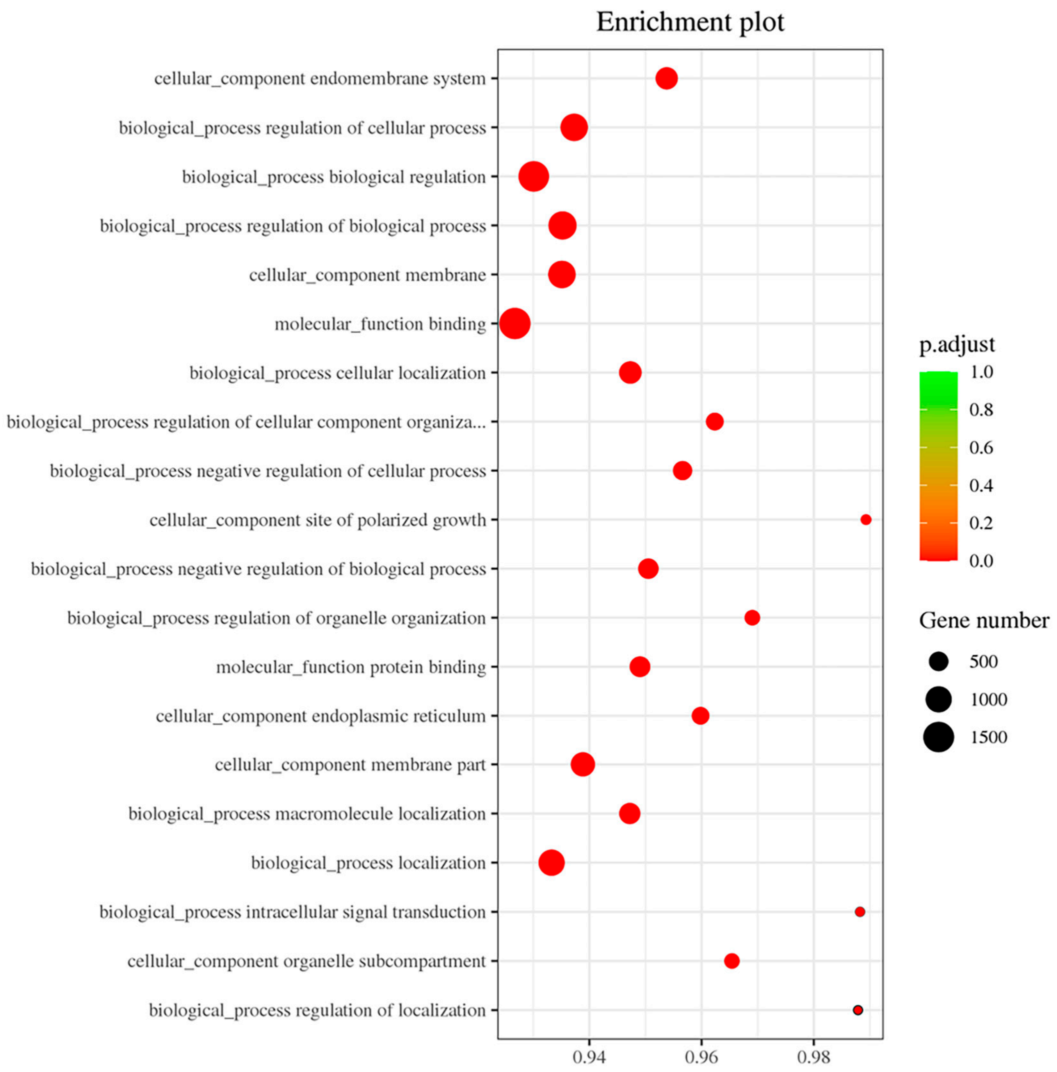

Figure S7. GO enrichment analyses of genes probably affected by CsAtf1.

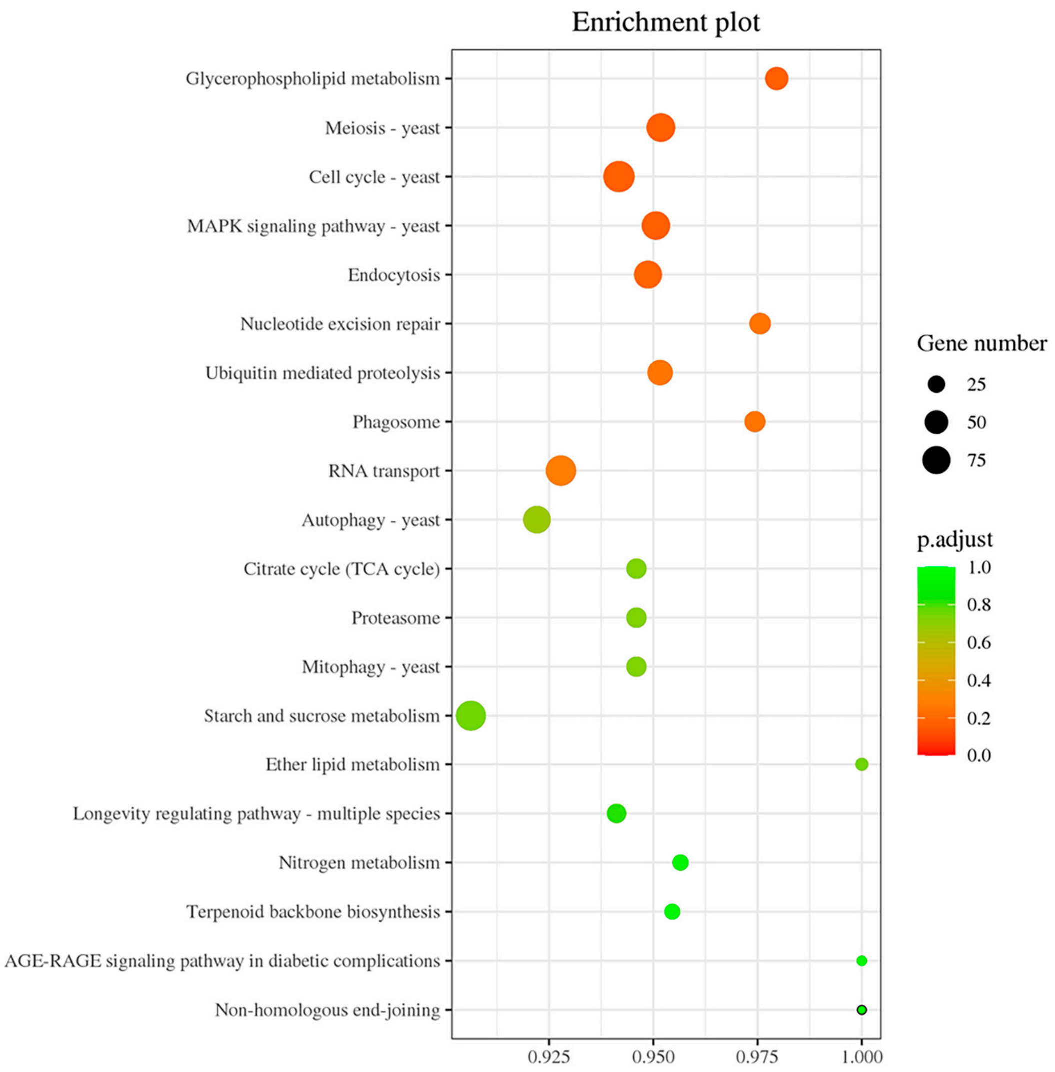

Figure S8. KEGG enrichment analyses of genes probably affected by CsAtf1.

Table S1. List of primers used in the study.

Primer name	sequence（5'- 3'）
CsCyp51G1-RT-F	AGAGTCGGGAGTTGGATTT
CsCyp51G1-RT-R	GCTGTTCTTTGCGTAGGAG
CsCyp51G1-1F	CAGACAACCGATGGAAGG
CsCyp51G1-2R	ATAGGCATTGATGTGTTGACCTCCAAAGTGGACTATGGAGGGAG
CsCyp51G1-3F	TATTGCACGGGAATTGCATGCTCTCACCCCGATGTAAGGTGCCAGTT
CsCyp51G1-4R	CCGCCCGTCTTTCGTCAA
CsCyp51G1-5F	ATGCGGGATATGCGAACGTTG
CsCyp51G1-6R	CTTCCTCGCTCACCTCATAGT
CsCyp51G1-OF	AGACAGGCACAGACAGACCACG
CsCyp51G1-OR	CTGCTACTCTCAACCTAACCAC
CsCyp51G1-pF	AAGTGACATGCTGACCATTCC
CsCyp51G1-pR	AGTAGCCACAACCAGGTCG
Q*CsCyp51G1*-Y1H-F	CGGAATTCGTGAGATTTAGGTCGGCATCG
Q*CsCyp51G1*-Y1H-R	CGAGCTCCTTTTCGTCTCAAAGTGGACT
CsAtf1-OF	ATGGGAACTTCGCCGACCGAC
CsAtf1-OR	TCATGAGAAACGTCGCTGGA
CsAtf1-Y1H-F	CGGAATTCATGGGAACTTCGCCGACCGAC
CsAtf1-Y1H-R	CGGGATCCTCATGAGAAACGTCGCTGGA
Actin-F	TGGTATGGGCCAGAAGGA
Actin-R	GGACGGAAGGAGCGAACA
RP27-F	TTTCGTAGGAACCCAATCTTCAAAATGGCCGACCCGTTTGCG
Stag-R	TACGTGCGCAGAGGAGCCTGAAT
S1F	GTGCCAACGCCACAGTGCCCCACA
S2R	R：GTGAGAGCATGCAATTCCCGTGCAATA
S2F	GGCGGTGCTATCCTTCCCGTGTT
S1R	GTTCAACGCCGCCTTCCGACAAAAT
M13F	TGTAAAACGACGGCCAGT
T7	TAATACGACTCACTATAGGG
pGADT7-F	TAATACGACTCACTATAGGGC
pGADT7-R	AGATGGTGCACGATGCACAG

Table S2. Cytochrome oxidase related genes that probably directly regulated by CsAtf1.

No.	Gene ID	Description	ChIP-Seq		RNA-Seq		
			Peak ID	Gene feature	Average-fpkm-WT	Average-fpkm-△*CsAtf1*	Diffexp-log2fc
1	43616755	CYP51; sterol 14alpha-demethylase	CsbZip_peak_781	CGMCC3_g555:exon	42.90	59.20	0.48
2	43615811	Cytochrome P450 monooxygenase 3	CsbZip_peak_657	CGMCC3_g470:exon	6.66	3.87	-0.75
3	43618144	COX7; cytochrome c oxidase subunit 7	CsbZip_peak_1059	CGMCC3_g680:Promoter	3747.92	2537.71	-0.54
4	43605293	Cytochrome P450 monooxygenase	CsbZip_peak_2249	CGMCC3_g1156:Promoter	3.68	1.51	-1.22
5	43613263	COX5B; cytochrome c oxidase subunit 5b	CsbZip_peak_4239	CGMCC3_g2406:intron	4256.31	2884.18	-0.54
6	43613986	QCR9, UCRC; ubiquinol-cytochrome c reductase subunit 9	CsbZip_peak_5482	CGMCC3_g3057:Promoter	761.65	611.62	-0.29
7	43613986	QCR9, UCRC; ubiquinol-cytochrome c reductase subunit 9	CsbZip_peak_5483	CGMCC3_g3057:Promoter	761.65	611.62	-0.29
8	43613694	SDHC, SDH3; succinate dehydrogenase (ubiquinone) cytochrome b560 subunit	CsbZip_peak_8251	del_diff	293.01	362.13	0.33
9	43615359	SDHC, SDH3; succinate dehydrogenase (ubiquinone) cytochrome b560 subunit	CsbZip_peak_8252	intergenic	293.01	362.13	0.33
10	43616258	CYC1, CYT1, petC;	CsbZip_peak_886	CGMCC3_g5101:Promoter	1092.01	855.45	-0.33

		ubiquinol-cytochrome c reductase cytochrome c1 subunit	3				
11	43616363	Cytochrome P450 52A13	CsbZip_peak_9101	intergenic	34.27	10.04	-1.73
12	43617987	Cytochrome	CsbZip_peak_12116	CGMCC3_g6659:exon	4.02	1.126	-1.72
13	43618357	fabF, OXSM, CEM1; 3-oxoacyl-[acyl-carrier-protein] synthase II	CsbZip_peak_12945	CGMCC3_g6992:exon	17.06	25.28	0.58
14	43619211	COX15, ctaA; cytochrome c oxidase assembly protein subunit 15	CsbZip_peak_14503	CGMCC3_g7760:exon	142.75	60.51	-1.21
15	43620252	CYP51, Eburicol 14-alpha-demethylase	CsbZip_peak_16380	CGMCC3_g8698:Promoter	42.90	59.20	0.48
16	43603622	SCS7, Ceramide very long chain fatty acid hydroxylase	CsbZip_peak_18191	CGMCC3_g10055:exon	437.22	462.13	0.07
17	43604191	Cytochrome P450 monooxygenase	CsbZip_peak_19191	intergenic	1.26	0.28	-1.87
18	43605291	QCR2, UQCRC2; ubiquinol-cytochrome c reductase core subunit 2;	CsbZip_peak_21219	CGMCC3_g11558:exon	813.70	611.28	-0.39
19	43605291	QCR2, UQCRC2; ubiquinol-cytochrome c reductase core subunit 2	CsbZip_peak_21220	CGMCC3_g11558:exon	813.70	611.28	-0.39
20	43605973	COX11, ctaG; cytochrome c oxidase assembly protein subunit 11	CsbZip_peak_22134	CGMCC3_g12171:Promoter	113.74	83.31	-0.43
21	43606092	Bifunctional cytochrome P450/NADPH--P450	CsbZip_peak_22324	CGMCC3_g12279:Promoter	12.76	22.86	0.83

		reductase					
22	43606687	COX5A; cytochrome c oxidase subunit 5a	CsbZip_peak_23063	CGMCC3_g12814:exon	1653.85	1248.59	-0.38
23	43606817	Cytochrome P450 61	CsbZip_peak_23378	CGMCC3_g12931:Promoter	342.05	281.13	-0.22
24	43610339	CYP55, fungal nitric oxide reductase	CsbZip_peak_28085	CGMCC3_g16101:Promoter	3.58	11.45	1.50
